# Response competition better explains Stroop interference than does response exclusion

**DOI:** 10.3758/s13423-020-01846-0

**Published:** 2020-11-24

**Authors:** Ardi Roelofs

**Affiliations:** Donders Institute for Brain, Cognition and Behaviour, Centre for Cognition, Radboud University, Spinoza Building B.02.30, Montessorilaan 3, 6525 HR Nijmegen, The Netherlands

**Keywords:** Articulatory buffer, Competition, Response exclusion, Stroop interference

## Abstract

Researchers debate whether Stroop interference from an incongruent word in color-naming response time is caused by response competition or by response exclusion. According to the former account, the interference reflects competition in lexical response selection during color name planning, whereas according to the latter, the interference reflects the removal of a motor program for the incongruent word from an articulatory buffer after planning. Here, numerical predictions about the magnitude of Stroop interference as a function of stimulus onset asynchrony were derived from these accounts. These predictions were then tested on representative data in the literature. Measures of goodness-of-fit showed that the numerical predictions of a response competition account are closer to the empirical data than those of the response exclusion account. These results indicate that response competition provides a better explanation of interference in naming than does response exclusion.

The color-word test designed by Stroop (1935) has become one of the most widely used tests in the cognitive and brain sciences. It is considered to be a gold standard of attentional measures (MacLeod, 1992), both in the laboratory and in the clinic. In modern computerized versions of the test, participants are instructed to vocally name the presentation color of printed incongruent color words (e.g., the word *green* printed in red ink; say “red”) or series of *X*s in a control condition. The stimuli are presented individually on a computer screen with Stroop condition randomized. Mean response time (RT) is typically longer on trials in the incongruent condition than in the control condition, which is called *Stroop interference* (see MacLeod, 1991, for a review).

According to a classic explanation, Stroop interference in RT is due to a response-related bottleneck caused by a response buffer that can hold only a single word (e.g., Morton, 1969; Morton & Chambers, 1973). As was first observed by Cattell (1886), reading is faster than color naming (about 100–200 ms; see M. O. Glaser & Glaser, 1982; W. R. Glaser & Düngelhoff, 1984). Therefore, the response that is elicited by the printed incongruent word will occupy the output buffer before the response obtained from the color, while in the control condition, only the color name response is available. Stroop interference is assumed to reflect the time it takes to remove the incongruent word from the buffer so that the name for the color can be produced. The account lost popularity after 1990, when several computationally implemented accounts were advanced that assume response competition during color name planning rather than clearing of a response buffer after planning as the cause of Stroop interference (e.g., Cohen, Dunbar, & McClelland, 1990; Roelofs, 2003). The response competition account holds that the two potential responses in the incongruent condition compete for selection during color name planning and slow responding down relative to the control condition, where only the color name response is available. Computer simulations showed that the assumption of response competition during color name planning explains many findings on Stroop interference. However, 15 years ago, the response buffer account of Stroop interference was revived, and the claim was made that it provides a better explanation for the interference than does the response competition account (e.g., Mahon, Garcea, & Navarrete, 2012).

Finkbeiner and Caramazza (2006) and Mahon, Costa, Peterson, Vargas, and Caramazza (2007) argued that Stroop interference in color naming reflects the time needed for excluding the articulatory program for the incongruent distractor word from a motor output buffer. They stated the following:

In the case of the Stroop and picture-word interference tasks, printed words, compared with colors or pictures, have privileged access to the articulators. . . . On this account, the target response (the picture or color name) can be produced only if the single-channel output buffer is not occupied by a representation corresponding to the distractor word. (Mahon et al., 2007, p. 524)

Interference may arise at the point of deciding which of two articulatory programs should be excluded from the output buffer in order that the correct response may be produced. (Finkbeiner & Caramazza, 2006, p. 1033)

Whether response exclusion provides a better explanation of Stroop interference than does response competition is a hotly debated issue (e.g., Kinoshita, De Wit, & Norris, 2017; Mahon et al., 2012; Mahon & Navarrete, 2014; Mulatti & Coltheart, 2012, 2014; Roelofs & Piai, 2013, 2015). The deeper issue at stake is whether lexical selection in spoken word production is by competition (e.g., La Heij, Kuipers, & Starreveld, 2006; Levelt, Roelofs, & Meyer, 1999; Roelofs, 1992; Roelofs, Piai, & Schriefers, 2011) or not (e.g., Dhooge & Hartsuiker, 2010, 2011, 2013; Finkbeiner & Caramazza, 2006). Given that the response exclusion account has never been computationally implemented (whereas response competition accounts were implemented; e.g., Cohen et al., 1990; Roelofs, 2003), previous tests between the two accounts concerned qualitative predictions about the presence or absence of effects in various Stroop conditions. However, as I make clear below, quantitative predictions can be derived from the response exclusion account, and these can be tested against existing data.

In what follows, numerical predictions are deduced from the response exclusion and response competition accounts about the magnitude of Stroop interference in color naming RT as a function of stimulus onset asynchrony (SOA). These numerical predictions were then tested on representative data in the literature, in particular, data from M. O. Glaser and Glaser (1982) and from Roelofs (2010).

## Numerical predictions by the response exclusion account

According to an influential psycholinguistic model (Levelt et al., 1999), naming a picture or color involves conceptual identification of the perceived stimulus (the color in the Stroop task), lexical response selection, and encoding of the word form, which includes morphological, phonological, and phonetic encoding, followed by articulation. In Roelofs (2003), I successfully applied this model to the Stroop task, assuming that competition in lexical response selection is the source of Stroop interference.

The articulatory buffering assumed by the response exclusion account can only happen after the preparation of an articulatory program in phonetic encoding, which has been estimated to start about 455 ms after stimulus onset in the case of a naming RT of 600 ms (Indefrey, 2011; Indefrey & Levelt, 2004). During phonetic encoding, articulatory programs are retrieved from long-term memory and may be placed in the articulatory buffer. Retrieval of the programs from memory takes some time. Thus, the articulatory buffer is reached somewhat later than the onset of phonetic encoding in color naming. If the buffer happens to be occupied by the articulatory program for the incongruent printed word, this program needs to be excluded. After the exclusion, some time is required to initiate articulation of the color name (cf. Meyer & Kieras, 1997). Quantitative predictions can be derived from the response exclusion account by considering the magnitude of Stroop interference in color naming as a function of SOA.

In a classic study, M. O. Glaser and Glaser (1982) examined the time course of Stroop interference by presenting incongruent words or neutral *X*s at a wide range of distractor preexposure SOAs (henceforth indicated by a minus sign) and postexposure SOAs. The printed stimuli were presented in white on a dark background, and the colors were presented as colored rectangles. The SOAs included all values differing by 100 ms between 400-ms distractor preexposure and 400-ms postexposure. Critical for testing the response exclusion account is that at the SOA of −400 ms, a 25-ms interference effect was obtained by Glaser and Glaser, while at the SOA of 0 ms, the magnitude of the interference was 72 ms. At the SOA of +200 ms, the interference was 24 ms. Later studies have obtained equivalent findings (e.g., W. R. Glaser & Glaser, 1989; Long & Lyman, 1987; Roelofs, 2010, 2014).

**Fig. 1:**
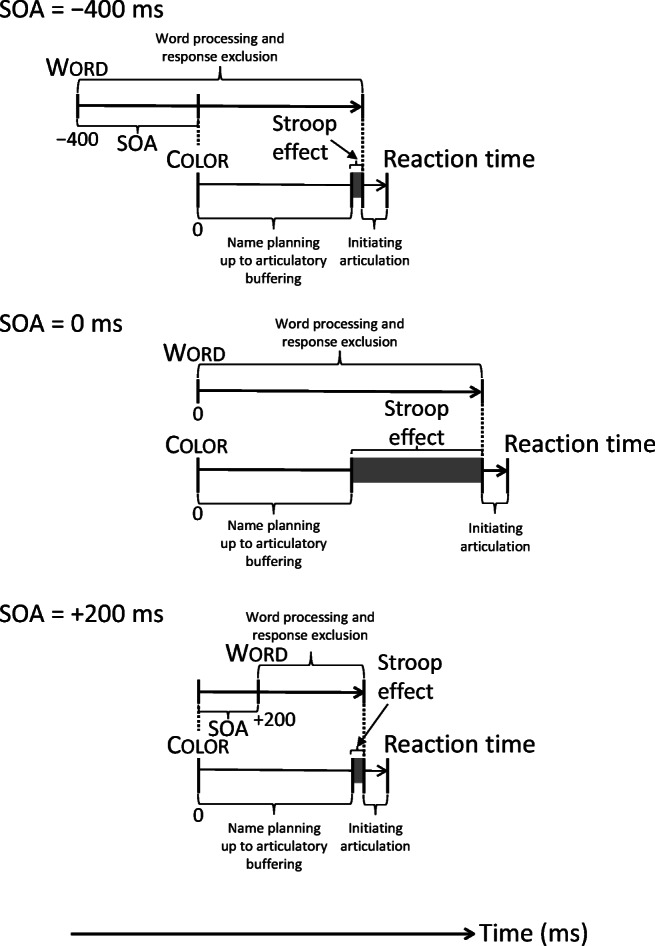
Time course of word and color processing as a function of stimulus onset asynchrony (SOA) in Stroop color naming under the response exclusion account. The magnitude of the Stroop effect at the SOAs of −400 ms and +200 ms is based on M. O. Glaser and Glaser (1982), and the magnitude of the effect at SOA = 0 ms is the prediction based on the effect at SOA = −400 ms

Figure 1 displays the time course of word processing and color name planning as a function of SOA under the response exclusion account. The figure shows what the magnitude of Stroop interference at SOA = 0 ms should be given the magnitude of the interference at the preexposure SOA of −400 ms. The magnitude of interference at zero SOA can be derived as follows. Let RT_pSOA,inc_ and RT_pSOA,ctr_ denote the mean color naming RT at a preexposure or postexposure SOA in the incongruent and the control conditions, respectively. For Stroop interference to occur at these SOAs (i.e., RT_pSOA,inc_ − RT_pSOA,ctr_ > 0), distractor word processing and response exclusion should not be completed when color name planning reaches articulatory buffering. The time between reaching the articulatory buffer and the completion of the exclusion process is the Stroop effect. To formalize the prediction by the response exclusion account for SOA = 0 ms, let RT_zeroSOA,inc_ and RT_zeroSOA,ctr_ denote the mean RT at zero SOA in the incongruent and the control conditions, respectively. If RT_pSOA,inc_ − RT_pSOA,ctr_ > 0,

1 $$ {\mathrm{RT}}_{\mathrm{zeroSOA},\mathrm{inc}}-{\mathrm{RT}}_{\mathrm{zeroSOA},\mathrm{ctr}}=\max \left(0,\left({\mathrm{RT}}_{\mathrm{pSOA},\mathrm{inc}}-{\mathrm{RT}}_{\mathrm{pSOA},\mathrm{ctr}}\right)-\mathrm{pSOA}\right). $$

In words, the magnitude of Stroop interference at zero SOA is null or larger (i.e., response exclusion has no effect or delays responding). In the latter case, interference at zero SOA equals the interference at the preexposure SOA plus the SOA value (i.e., minus the negative SOA). At preexposure SOAs, distractor word processing and response exclusion have a head start compared with at zero SOA. As a consequence, the interference at zero SOA will be increased relative to the interference at the preexposure SOA. Using a postexposure SOA as the basis of the prediction, interference at zero SOA equals the interference at the postexposure SOA minus the SOA value. At postexposure SOAs, distractor word processing and response exclusion are delayed compared with at zero SOA. As a consequence, the interference at zero SOA will be decreased relative to at the postexposure SOA.

The top panel of Fig. 1 shows the time courses of distractor word and color processing at SOA = −400 ms, where interference was 25 ms. The middle panel shows the time courses of distractor word and color processing at SOA = 0 ms. Distractor word processing and response exclusion here have no head start of 400 ms. Consequently, the initiation of articulation will be delayed by an extra 400 ms. The magnitude of Stroop interference should now be 25 ms − −400 ms = 425 ms. This is much larger than the 72 ms observed by M. O. Glaser and Glaser (1982). The bottom panel shows the time courses of distractor word and color processing at SOA = +200 ms. At this SOA, the interference was 24 ms. At SOA = 0 ms, distractor word processing and response exclusion start 200 earlier, so that the interference is predicted to be 24 ms − +200 ms = −176 ms, implying that the initiation of articulation should no longer be delayed. However, Glaser and Glaser observed 72-ms interference at zero SOA.

Articulatory buffering happens during phonetic encoding in color name planning. Thus, assuming an onset of phonetic encoding at 455 ms after color presentation onset (cf. Indefrey, 2011) underestimates the moment that the articulatory buffer is reached. Consequently, a 25-ms interference effect at SOA = −400 ms means that distractor word processing and response exclusion took longer than 400 ms (preSOA) + 455 ms (color name planning up to phonetic encoding, underestimating buffering onset) + 25 ms (the Stroop effect at the preSOA) = 880 ms. This seems unreasonably long for processing and excluding a motor program for a printed word from an articulatory buffer, given that oral word reading in the study of M. O. Glaser and Glaser (1982) took on average about 430 ms.

The response exclusion hypothesis was originally developed to account for data from the picture-word equivalent of the Stroop task (e.g., Dhooge & Hartsuiker, 2010, 2011, 2013; Finkbeiner & Caramazza, 2006). In the picture-word task, participants have to name pictured objects (e.g., a picture of a cat; say “cat”) while trying to ignore superimposed printed distractor words, which may be semantically related (e.g., *dog*) or unrelated (e.g., *house*), or they see a series of *X*s in the control condition (W. R. Glaser & Düngelhoff, 1984). The semantically related condition is taken to be the equivalent of the incongruent condition in the color-word Stroop task. In both conditions, an incongruent distractor word is presented from the same semantic category as the target word, like distractor *dog* for the target “cat” and the distractor *green* for the target “red.” To examine whether the response exclusion account correctly predicts the magnitude of the Stroop-like effect in picture-word interference (i.e., the difference in naming RT between semantically related distractors and series of *X*s), I derived predictions for the classic data of W. R. Glaser and Düngelhoff (1984). They examined the time course of picture-word interference by presenting incongruent (semantically related) words or *X*s. The SOAs included all values differing by 100 ms between 400 ms distractor preexposure and 400 ms postexposure. At SOA = −400 ms, no interference was observed, but at SOA = −300 ms, a 69-ms effect was obtained. Based on this effect, the response exclusion account predicts an interference effect of 369 ms for SOA = 0 ms. However, empirically, the magnitude of the interference was 131 ms, which is again much smaller than predicted. Thus, also for picture-word interference, the numerical predictions by the response exclusion account do not hold.

In the studies of M. O. Glaser and Glaser (1982) and W. R. Glaser and Düngelhoff (1984), naming trials were blocked by SOA. For example, in a block of trials in the color-word Stroop study of Glaser and Glaser, the SOA was always −400, 0, or +200 ms. Perhaps participants have exploited the preknowledge about the SOA. It is possible that they adopted a more relaxed mode of response exclusion at the SOA of −400 ms than at 0 ms. If so, response exclusion may have happened quicker at zero SOA than predicted on the basis of the preexposure SOA. Similarly, they may have adopted a more relaxed mode of response exclusion at the SOA of 0 ms than at +200 ms. In Roelofs (2010), I reported Stroop experiments in which SOAs were either constant or variable across trials, and with interstimulus intervals (ISIs) being either constant or variable across trials. With variable SOAs and variable ISIs, a block-wide strategy of relaxing response exclusion cannot be adopted. Below, I report a test of the numerical predictions by the response exclusion account using these data.

## Numerical predictions by the response competition account

Earlier, I indicated that the response exclusion account of Stroop interference had lost popularity 3 decades ago, when computationally implemented accounts were proposed that assume competition in response selection as the cause of Stroop interference. In Roelofs (2003), I reported the results of computer simulations with such a response competition model, called WEAVER++, which implements the psycholinguistic theory of word planning advanced by Levelt et al. (1999). The simulations revealed a quantitative fit between the model and a wealth of data on Stroop task performance, including the effect of SOA on Stroop interference observed by M. O. Glaser and Glaser (1982) and on picture-word interference observed by W. R. Glaser and Düngelhoff (1984). Moreover, in Roelofs (2018), I reported simulation results showing that the model accounts for the cumulative interference in continuous picture naming and interference in blocked-cyclic picture naming.

Below, I also report a test of the numerical predictions by WEAVER++ using the Stroop interference data of Roelofs (2010). These numerical predictions were made without estimating any new parameter values (originally estimated for the data of M. O. Glaser & Glaser, 1982).

## Method

I tested numerical predictions by the response exclusion and response competition accounts on the data reported in Roelofs (2010). These data were used for four reasons. First, the data are representative in that they show the same pattern of Stroop interference across SOAs as in other studies in the literature (i.e., M. O. Glaser & Glaser, 1982; Long & Lyman, 1987; Roelofs, 2014). Second, the data include Stroop interference at zero SOA as well as at the long preexposure SOA of −400 ms, which provides the strongest test of the predictions of the response exclusion account. Except for the experiment of Glaser and Glaser, other RT studies in the literature (i.e., Long & Lyman, 1987; Roelofs, 2014) do not have the SOA of −400 ms. Third, I have access to the raw RT data of this study, but not of the other studies (i.e., Glaser & Glaser, Long & Lyman) to compute a statistical measure of goodness-of-fit between predictions and the real data. Fourth, my study employs a randomization of SOAs and ISIs across trials, which prevents possible processing strategies that may otherwise be assumed to save the response exclusion account.

The data sets reported in Roelofs (2010) provide evidence on the magnitude of Stroop interference at SOAs including −400, 0, and +200 ms with trials either blocked by SOA or SOA randomly varying, and ISI being either constant or randomly varying, hereafter referred to as the *randomness condition*. For the response exclusion account, I used the empirically observed interference at SOA = −400 ms to predict the effect at SOA = 0 ms using Eq. 1, above. For WEAVER++, I used the numerical predictions made for SOA = 0 ms of M. O. Glaser and Glaser (1982). The predictions of the two accounts were evaluated using 95% confidence intervals (Cumming, 2014), mean absolute errors (Willmott & Matsuura, 2005), and a goodness-of-fit chi-square test for models of latency (Miller & Greeno, 1978), whereby a large and significant chi-square indicates a poor fit. The RT data and tests are available from the archive of the Open Science Framework (https://osf.io/pfvgw).

## Results

Table 1 shows the predicted and empirically observed magnitudes of Stroop interference at SOA = 0 ms. The table reveals that the numerical predictions by the response exclusion account, but not those by the response competition account, all fall outside the 95% confidence intervals for the observed effects. The mean absolute error, the average of the absolute differences between predicted and empirical effects, is 333 ms for the response exclusion account and 13 ms for the response competition account. The goodness-of-fit chi-square test for the numerical predictions by the response exclusion account showed that χ^2^ = 2583.97, *p* < .0001, which indicates that the predictions do not agree with the empirical observations. For the predictions of the response competition account, χ^2^ = 5.03, *p* = .28, which indicates that the numerical predictions do not statistically differ from the empirical observations. Based on a failure to reject a null hypothesis of no misfit, one cannot conclude that a particular model is good. Still, the difference in misfit allows one to conclude that the competition account does better than the exclusion account. Thus, in terms of 95% confidence intervals, mean absolute errors, and chi-square tests, the response competition account better fits the empirical data than does the response exclusion account.

**Table 1:** Predictions by the response exclusion and response competition accounts and empirically observed Stroop interference effects

Randomness condition	Exclusion	Competition	Observed	95% CI
Constant SOA and constant ISI	435	97	117	[95, 138]
Variable SOA and constant ISI	442	97	111	[79, 142]
Constant SOA and variable ISI	448	97	104	[77, 130]
Variable SOA and variable ISI	448	97	108	[83, 133]

*Note*. 95% CI = 95% confidence interval for the observed effect. SOA = stimulus onset asynchrony; ISI = interstimulus interval. Predicted and observed effects are in milliseconds. The observed effects are from Roelofs (2010)

## Discussion

I derived numerical predictions from the response exclusion and response competition accounts about the magnitude of Stroop interference as a function of SOA, and then tested these predictions using representative data in the literature (i.e., Roelofs, 2010). Confidence intervals, mean absolute errors, and chi-square tests revealed that the predictions of the response competition account are closer to the empirical data than the predictions of the response exclusion account.

To save their account in the face of the misfit of the SOA effects in color-word Stroop and picture-word interference, proponents of the response exclusion account might argue that the conjectured different degrees of relaxation of response exclusion happen on a trial-by-trial basis rather than block wide. Each degree of relaxation would be such that it exactly fits the data. However, response exclusion would than become like the dragon in Carl Sagan’s garage (also known as Russell’s teapot). When you ask him why this dragon cannot be seen, he responds that it is an invisible dragon; when asked why the heat cannot be detected by an infrared sensor, he says that the dragon spits heatless fire; and so on. Sagan (1996) states,

Now, what’s the difference between an invisible, incorporeal, floating dragon who spits heatless fire and no dragon at all? If there’s no way to disprove my contention, no conceivable experiment that would count against it, what does it mean to say that my dragon exists? (p. 171)

Clearly the “no dragon” explanation is to be preferred over the assumption of the existence of a dragon, and the same holds for “no response exclusion” in light of the evidence reported in the present article.

I also tested the numerical predictions of the WEAVER++ model, which implements a response competition account, on the Stroop interference data of Roelofs (2010). Without estimating any new parameter values, the magnitude of the predicted Stroop interference was close to that of the observed effects.

To conclude, representative data on SOA effects are better fit by a response competition account than by the response exclusion account of Stroop interference. These results indicate that response competition provides a better account of interference in naming than does response exclusion, and they suggest that lexical selection in word production is more satisfactorily explained by competition than by no competition.

### Author note

I am indebted to Sachiko Kinoshita, Colin M. MacLeod, and Joachim Vandekerckhove for helpful comments.

### Open practices statement

The data are available from the archive of the Open Science Framework (osf.io/pfvgw).
